# Associations of COVID-19-Related Health, Healthcare and Economic Factors With Prenatal Depression and Anxiety

**DOI:** 10.3389/ijph.2022.1604433

**Published:** 2022-05-04

**Authors:** Lyndsay A. Avalos, Nerissa Nance, Sylvia E. Badon, Kelly Young-Wolff, Jennifer Ames, Yeyi Zhu, Monique M. Hedderson, Assiamira Ferrara, Ousseny Zerbo, Mara Greenberg, Lisa A. Croen

**Affiliations:** Division of Research, Kaiser Permanente Northern California, Oakland, CA, United States

**Keywords:** perinatal, mental health, psychological distress, mood disorder, pregnancy

## Abstract

**Objective:** This study evaluated whether COVID-19 pandemic-related health, healthcare and economic factors during pregnancy are associated with prenatal depression and anxiety.

**Methods:** We conducted a cross-sectional study of 6,628 pregnant members of Kaiser Permanente Northern California who responded to a survey between 22 June and 30 September 2020. The survey included questions about depression (Patient Health Questionnaire) and anxiety (Generalized Anxiety Disorder) symptoms and COVID-19-related health and healthcare (e.g., had COVID-19) and economic (e.g., food insecurity) factors.

**Results:** Over one third of individuals reported depression (25% mild, 8% moderate, 3% severe) or anxiety (22% mild, 8% moderate, 5% severe) symptoms. In multivariable analyses, COVID-19 during pregnancy, employment with greater risk of COVID-19, distress over changes in prenatal care, job loss, changes in childcare and food insecurity were associated with greater odds of prenatal depression or anxiety.

**Conclusion:** Findings suggest the COVID-19 pandemic may have severe mental health repercussions for pregnant individuals. Support services for pregnant individuals experiencing these COVID-19-related factors and monitoring of those who had moderate/severe prenatal depression and anxiety symptoms during the COVID-19 pandemic is warranted.

## Introduction

The coronavirus pandemic (COVID-19) has had a tremendous impact worldwide. At the time of publication of this paper, there have been >76 million confirmed cases and nearly 900,000 deaths in the US alone (1). While these data demonstrate the enormous impact on morbidity and mortality directly related to infection, the pandemic has triggered social, behavioral and economic changes that may also have psychological effects. Since the pandemic has started, an increasing amount of research has documented the mental health impacts of the COVID-19 pandemic, including on depression and anxiety (2–4).

The potential impact of the COVID-19 pandemic on the mental health of pregnant individuals is important to understand. Pregnancy is an extremely vulnerable period given the increased susceptibility to adverse health effects for both the mother and offspring. Pregnant individuals have experienced distinct concerns and challenges during the pandemic. Concerns about fewer prenatal visits, cancellation of labor and delivery classes, virtual visits, and the possibility of heightened risks of adverse pregnancy and child outcomes associated with COVID-19 (5) may lead to increases in psychological distress for pregnant individuals. In addition, a household member with COVID-19 and employment that may increase the risk of exposure to infection may have a similar impact on prenatal psychological distress. The stay-at-home orders and shelter-in-place advisories implemented to control and reduce the spread of the virus (6) not only resulted in physical and social isolation from family and friends but also led to business closures and the highest US unemployment rate recorded (7). Loss of employment by a pregnant woman or her partner when the need for economic security is great may have a significant impact on her mental health (8). It may also lead to other stressors such as food insecurity (9) that may increase psychological distress (9). The closure of schools and day cares left families who did not lose their jobs struggling to find childcare and often facing dramatic increases in childcare costs. These COVID-19-related health, healthcare and economic factors are all likely to compound psychological distress specifically depression and anxiety in pregnant individuals.

Depression and anxiety during pregnancy can have significant negative consequences for both the parent and baby (10–16). Thus, there is a need to understand the most salient COVID-19-related factors contributing to prenatal depression and anxiety to prevent the emergence of severe mental illness and identify areas for targeted interventions and treatments. This study evaluates associations between COVID-19-related health, healthcare changes and economic factors with anxiety and depression symptoms in pregnant individuals seeking prenatal care in Kaiser Permanente Northern California (KPNC), a large integrated healthcare delivery system.

## Methods

### Setting and Study Design

This cross-sectional study was conducted in Kaiser Permanente Northern California (KPNC), a large integrated health care delivery system that provides comprehensive medical care to over 4.5 million members. All 15 regional service centers (with 48 associated office facilities) have Obstetrics and Gynecology and Behavioral Medicine/Psychiatry Departments. KPNC members include individuals covered by employer-sponsored insurance plans, the insurance exchange and Medicaid. Coverage is provided for approximately 30% of the northern California population. KPNC members are socio-demographically similar to the population living in the geographic area (17). KPNC maintains comprehensive electronic health records (EHR).

Beginning on June 22, 2020 pregnant KPNC members were invited to participate in a KPNC COVID-19 Pregnancy Study (18). The survey collected information on depression and anxiety symptoms, as well as COVID-19-related health, healthcare and economic factors. Pregnant individuals 18 years of age or older and English-speaking were identified from the EHR and recruited via email. At the initial survey launch, all eligible individuals were contacted via email and invited to complete a brief, web-based survey. Every 2 weeks thereafter, all newly eligible individuals were emailed a study invitation. Individuals who did not respond to the initial email invitation received email reminders 7 and 14 days after the initial emails were sent. Participants who started but did not complete the survey received two email reminders, at 7 and 14 days after starting the survey. While study recruitment is ongoing, this study reports on data collected from 6,628 individuals who were at any stage of pregnancy between 1 January 2020 through 30 September 2020.

The Institutional Review Board of KPNC approved all study procedures and individuals indicated informed consent by completing the survey after reviewing the consent information in the recruitment email (protocol number 1600113-11).

### Measures

#### Outcomes

Depression: The Patient Health Questionnaire (PHQ-8) (18) is a depression screener that has been validated in perinatal populations (19, 20) with high sensitivity (77%) and specificity (62%) (19) and was used to assess depression symptoms in the past 2 weeks. Scores range from 0 to 24 and were categorized as 1-4 (none to minimal depression), 5-9 (mild depression ), 10-14 (moderate depression), and 15-24 (severe depression).

Anxiety: General Anxiety Disorder Scale (GAD-7) (21) has been validated in prenatal populations (22) with good sensitivity (76%) and specificity (73%) (22) and was used to measure anxiety symptoms in the past 2 weeks. Scores range from 0 to 21 and are categorized as minimal/none (0–4), mild (5–9), moderate (10–14), and severe (15–20) (23) anxiety.

#### Exposures

Health and healthcare factors: Individuals were asked if a healthcare provider had told them that they had or likely had COVID-19 during their pregnancy (COVID-19 in pregnancy; y/n); if a member of their household had or probably had COVID-19 (Household member had COVID-19; y/n); whether their job put them at increased risk of COVID-19 (high-risk employment; y/n); and how distressed they were about changes to their prenatal care due to the COVID-19 pandemic (moderately/extremely vs. not at all/mildly).

Economic factors: All questions addressing economic factors referred to the time frame of “since becoming aware of the COVID-19 pandemic”. Individuals were asked if they lost their job permanently, temporarily or reduced their work hours (lost job/reduced hours; y/n), if their spouse lost their job permanently, temporarily or reduced their work hours (partner lost job/reduced hours; y/n), if their childcare was impacted such that they had difficulty arranging childcare or had to pay more (childcare challenges; y/n). The validated 2-item Hunger Vital Sign screener was used to assess food insecurity (24). Food insecurity (y/n) was defined as responding often true or sometimes true (vs. never true) to either 1) worried that food would run out before getting more money or 2) the food they bought did not last and they didn’t have enough money to get more.

#### Covariates

Demographic and health characteristics were ascertained from KPNC’s electronic health records (EHR) and include: race/ethnicity (Black, Latina, Asian, Other, White) maternal age at delivery (<25, 25–29, 30–34, 35+ years), insurance status (Medicaid/public, private), trimester at survey completion (trimester one/ two or three), parity (nulliparous, one, 2+ previous births), and history of a depression or anxiety disorder in the 3 years prior to pregnancy [anxiety (ICD-10 codes: F06.4, F40, F41, F42, F43.22, F43.23, O99.34) or depression (ICD-10 codes: F32.0-4,F32.8-9, F33.0-3, F34.1, F43.21, F43.23, F53.0, O90.6, O99.34)].

### Statistical Analysis

Inverse probability weighting was used to account for survey non-response (21% response rate). Inverse probability weights for survey response were calculated using predicted probabilities from a logistic regression model including variables expected to be associated with survey response (race/ethnicity category, insurance type, parity, history of depression or anxiety diagnosis, trimester of pregnancy at the start of the COVID-19 pandemic), additional variables strongly associated with depression or anxiety symptoms (maternal age), and an interaction term for race/ethnicity category and insurance type. Weights ranged from 1.74 to 40.24 in individuals who completed the survey. For the 14 individuals with weights greater than 20, weights were truncated at 20.

Weighted percentages were calculated for all categorical measures overall and by depression and anxiety severity status. For individuals with missing data for the exposure or covariates we used multiple imputation by chained equations (MICE) (25) with 100 imputations to impute missing values. After imputation, weighted multinomial logistic regression models (using the glogit option in the SAS logistic PROC) were run for each imputed dataset excluding participants with missing outcomes values for the respective models (*n* = 36 for prenatal depression and *n* = 44 for prenatal anxiety). Results were combined using Rubin’s rules (26). Crude point and interval estimates of odds ratios of depression (severe, moderate, mild vs. none) and anxiety (severe, moderate, mild vs. none) associated with each COVID-19-related factor were conducted. Adjusted models included all covariates (maternal age, parity, insurance status, history of a depression or anxiety disorder, race/ethnicity, and trimester at survey completion) and COVID-19-related factors. Analyses were conducted in SAS version 9.4 (SAS Institute INC, Cary NC).

## Results

Of the individuals in our study, 66% were 30 years of age or older, 8% had Medicaid insurance, and 18% had a history of depression or anxiety. Individuals reported several COVID-19 pandemic-related health and healthcare factors (2% reported COVID-19 infection during pregnancy, 4% reported a household member who had COVID-19 infection, 16% reported high-risk employment, 26% reported moderate/extreme distress related to changes in prenatal care) and economic factors (23% lost their job, 23% reported that a partner lost their job, 26% reported childcare challenges, and 19% reported food insecurity) (Table 1).

**TABLE 1:** Demographic characteristics and COVID-19-related factors of the sample, overall and by depression severity.

	Depression				
	Total	None	Mild	Moderate	Severe
	n (weighted column %)	n (weighted row %)	n (weighted row %)	n (weighted row %)	n (weighted row %)
Overall	6592 (100)	4338 (64)	1597 (25)	477 (8)	180 (3)
Characteristics					
Maternal age (years)					
<25	326 (8)	150 (47)	104 (30)	42 (14)	30 (9)
25–29	1332 (25)	841 (62)	316 (24)	125 (10)	52 (4)
30–34	2839 (41)	1951 (67)	658 (24)	179 (7)	51 (2)
35+	2093 (25)	1397 (67)	518 (25)	131 (6)	47 (2)
Missing	2 (0.1)	1 (50)	1 (50)	0 (0)	0 (0)
Race/ethnicity					
Black	242 (7)	125 (50)	71 (30)	27 (12)	19 (9)
Latina	1333 (27)	848 (62)	316 (24)	122 (10)	47 (4)
Asian	1442 (24)	988 (68)	331 (23)	93 (7)	30 (2)
Other	104 (2)	59 (54)	31 (33)	8 (8)	6 (5)
White	3222 (35)	2164 (67)	776 (24)	208 (7)	74 (3)
Missing	249 (5)	154 (60)	72 (30)	19 (7)	4 (2)
Trimester at survey completion					
1st/2nd	4185 (67)	2732 (43)	1027 (17)	313 (6)	113 (2)
3rd	2407 (33)	1606 (21)	570 (8)	164 (2)	67 (1)
Parity					
0	2027 (25)	1358 (16)	498 (6)	122 (2)	49 (1)
1	2074 (32)	1384 (21)	470 (7)	157 (3)	63 (1)
2+	897 (14)	588 (9)	213 (3)	69 (1)	27 (0.5)
Missing	1594 (29)	1008 (18)	416 (8)	129 (3)	41 (1)
Insurance status					
Medicaid/Public	311 (8)	173 (5)	89 (2)	31 (1)	18 (1)
Private	6223 (90)	4131 (59)	1490 (22)	442 (7)	160 (3)
Unknown	58 (2)	34 (1)	18 (58)	5 (0.1)	2 (0.1)
History of a depression or anxiety disorder					
Yes	1307 (18)	686 (9)	396 (5)	148 (2)	77 (1)
No	5285 (82)	3652 (55)	1201 (19)	329 (6)	103 (2)
COVID-19 pandemic-related Factors					
Health and Healthcare Factors					
COVID-19 in pregnancy					
Yes	108 (2)	60 (49)	24 (24)	15 (16)	9 (11)
No	6484 (98)	4278 (64)	1573 (25)	462 (8)	171 (3)
Household member had COVID-19					
Yes	242 (4)	133 (51)	70 (28)	25 (12)	14 (8)
No	6350 (96)	4205 (65)	1527 (24)	452 (8)	166 (3)
High-risk employment					
Yes	1023 (16)	573 (54)	286 (28)	99 (10)	65 (8)
No	5569 (84)	3765 (66)	1311 (24)	378 (8)	115 (3)
Distress due to prenatal care changes					
Moderately/Extremely	1676 (26)	828 (47)	532 (32)	212 (14)	104 (7)
Mildly/Not at all	4165 (62)	3011 (71)	899 (22)	206 (6)	49 (1)
Missing	751 (12)	499 (64)	166 (22)	59 (9)	27 (5)
Economic Factors					
Lost job					
Yes	1409 (23)	823 (56)	394 (29)	129 (10)	63 (6)
No	5183 (77)	3515 (67)	1203 (23)	348 (7)	117 (3)
Partner lost job					
Yes	1464 (23)	888 (58)	391 (28)	122 (9)	63 (5)
No	5128 (77)	3450 (66)	1206 (24)	355 (8)	117 (3)
Childcare challenges					
Yes	1750 (26)	1080 (60)	462 (26)	148 (9)	60 (5)
No	4778 (73)	3209 (65)	1125 (24)	325 (8)	119 (3)
Missing	67 (1)	49 (73)	10 (17)	4 (8)	1 (2)
Food Insecurity					
Yes	964 (19)	424 (43)	305 (32)	143 (15)	92 (10)
No	5605 (81)	3896 (69)	1288 (23)	334 (7)	87 (2)
Missing	23 (0.4)	18 (81)	4 (15)	0 (0)	1 (4)

Supplemental Table S1 documents several sociodemographic differences between study participants and non-responders. Compared to individuals who did not complete the survey, those who did were more likely to be White (51% vs. 33%), less likely to have Medicaid (8% vs. 10%), more likely to be in their first pregnancy (37% vs. 29%), and less likely to be in their first trimester of pregnancy when the survey was completed (12% vs. 30%). There were no differences by history of depression or anxiety.

### Prevalence of Depression and Anxiety Symptom Severity

Overall, 36% of the individuals reported depression symptoms (mild: 25%, medium: 8%, severe: 3%; Table 1) and 35% reported anxiety symptoms (mild: 22%, moderate: 8%, and severe 5%; Table 2). A quarter (25%, *n* = 1,559) of the individuals reported mild to severe symptoms of both depression and anxiety.

**TABLE 2:** Demographic characteristics and COVID-19-related factors of the sample overall and by anxiety severity.

	Anxiety				
	Total	None	Mild	Moderate	Severe
	n (weighted column %)	n (weighted row %)	n (weighted row %)	n (weighted row %)	n (weighted row %)
Overall	6584 (100)	4439 (65)	1469 (22)	461 (8)	215 (3)
Characteristics					
Maternal age					
<25	327 (8)	167 (51)	82 (24)	58 (19)	20 (6)
25–29	1331 (25)	879 (65)	265 (20)	115 (9)	72 (6)
30–34	2837 (41)	1960 (69)	650 (22)	158 (6)	69 (3)
35+	2087 (25)	1431 (69)	472 (23)	130 (6)	54 (2)
Missing	2 (0.1)	2 (100)	0 (0)	0 (0)	0 (0)
Race/ethnicity					
Black	243 (7)	132 (52)	61 (26)	30 (13)	20 (9)
Latina	1333 (27)	846 (63)	295 (22)	132 (11)	60 (5)
Asian	1435 (24)	1039 (73)	290 (20)	75 (5)	31 (2)
Other	104 (2)	64 (59)	25 (27)	12 (11)	3 (3)
White	3222 (35)	2180 (68)	751 (23)	197 (6)	94 (3)
Missing	247 (5)	178 (71)	47 (20)	15 (6)	7 (2)
Trimester at survey completion					
1st/2nd	4181 (67)	2857 (67)	910 (21)	286 (8)	128 (4)
3rd	2403 (33)	1582 (65)	559 (24)	175 (8)	87 (4)
Parity					
0	2024 (25)	1375 (67)	451 (23)	144 (8)	54 (3)
1	2070 (32)	1372 (65)	487 (23)	135 (7)	76 (4)
2+	897 (14)	591 (66)	201 (22)	65 (8)	40 (5)
Missing	1593 (29)	1101 (68)	330 (20)	117 (9)	45 (3)
Insurance Status					
Medicaid/Public	311 (8)	189 (60)	73 (24)	26 (8)	23 (8)
Private	6215 (90)	4217 (67)	1380 (22)	430 (8)	188 (3)
Unknown	58 (2)	33 (55)	16 (28)	5 (10)	4 (6)
History of a depression or anxiety disorder					
Yes	1306 (18)	662 (48)	393 (30)	158 (13)	93 (9)
No	5278 (82)	3777 (70)	1076 (20)	303 (7)	122 (3)
Health and Healthcare Factors					
COVID-19 in pregnancy					
Yes	108 (2)	59 (48)	33 (32)	8 (7)	8 (13)
No	6476 (98)	4380 (67)	1436 (22)	453 (8)	207 (4)
Household member had COVID-19					
Yes	244 (4)	151 (59)	64 (26)	19 (9)	10 (6)
No	6340 (96)	4288 (67)	1405 (22)	422 (8)	205 (4)
High-risk employment					
Yes	1021 (16)	580 (55)	246 (24)	123 (13)	72 (8)
No	5563 (84)	3859 (69)	1223 (22)	338 (7)	143 (3)
Distress due to prenatal care changes					
Moderately/Extremely	1676 (26)	819 (47)	500 (29)	223 (14)	134 (9)
Mildly/Not at all	4166 (62)	3134 (75)	798 (19)	181 (5)	53 (1)
Missing	742 (12)	486 (64)	171 (23)	57 (8)	28 (5)
Economic Factors					
Lost job					
Yes	1408 (23)	873 (61)	345 (24)	122 (10)	68 (5)
No	5176 (77)	3566 (68)	1124 (21)	339 (7)	147 (3)
Partner lost job					
Yes	1461 (23)	899 (60)	365 (25)	126 (10)	71 (5)
No	5123 (77)	3540 (68)	1104 (21)	335 (7)	144 (3)
Childcare challenges					
Yes	1746 (26)	1078 (60)	462 (26)	128 (8)	78 (5)
No	4775 (73)	2214 (69)	995 (20)	332 (8)	134 (3)
Missing	63 (1)	47 (73)	12 (20)	1 (1)	3 (6)
Food Insecurity					
Yes	960 (19)	453 (47)	280 (29)	130 (13)	97 (11)
No	5602 (81)	3967 (71)	1187 (20)	331 (7)	117 (2)
Missing	22 (0.3)	19 (88)	2 (9)	0 (0)	1 (4)

### COVID-19-Related Health, Healthcare and Economic Factors and Depression and Anxiety

Health and healthcare factors: Individuals who reported having COVID-19 during pregnancy were more likely than individuals who did not to report moderate and severe depression (moderate: 16% vs. 8%, severe: 11% vs. 3%) and severe anxiety symptoms (13% vs. 4%) (Tables 1, 2, respectively). Compared to individuals without high-risk employment, individuals with high-risk employment were more likely to have severe depression (8% vs. 3%) and moderate (13% vs. 7%) or severe (8% vs. 3%) anxiety. Compared to individuals who reported mild/no distress due to prenatal care changes, individuals who reported moderate/extreme distress were more likely to report depressive symptoms across all levels of severity (mild: 32% vs. 22%, moderate: 14% vs. 6%, severe: 7% vs. 1%) and anxiety (mild: 29% vs. 19%, moderate: 14% vs. 5%, severe: 9% vs. 1%).

Economic Factors: Although individuals impacted by pandemic-related economic factors (job loss, partner job loss, and childcare) had a higher prevalence of depression and anxiety overall compared to individuals not impacted by these factors there were few differences by severity (Tables 1, 2). However, compared to individuals with no food insecurity, individuals with food insecurity were more likely to have higher depression severity (mild: 32% vs. 23%, moderate: 15% vs. 7%, severe: 10% vs. 2%) and anxiety severity (mild: 29% vs. 20%, moderate: 13% vs. 7%, severe: 11% vs. 2%).

### Associations Between COVID-19-Related Health, Healthcare Changes and Economic Factors and Depression and Anxiety

**FIGURE 1:**
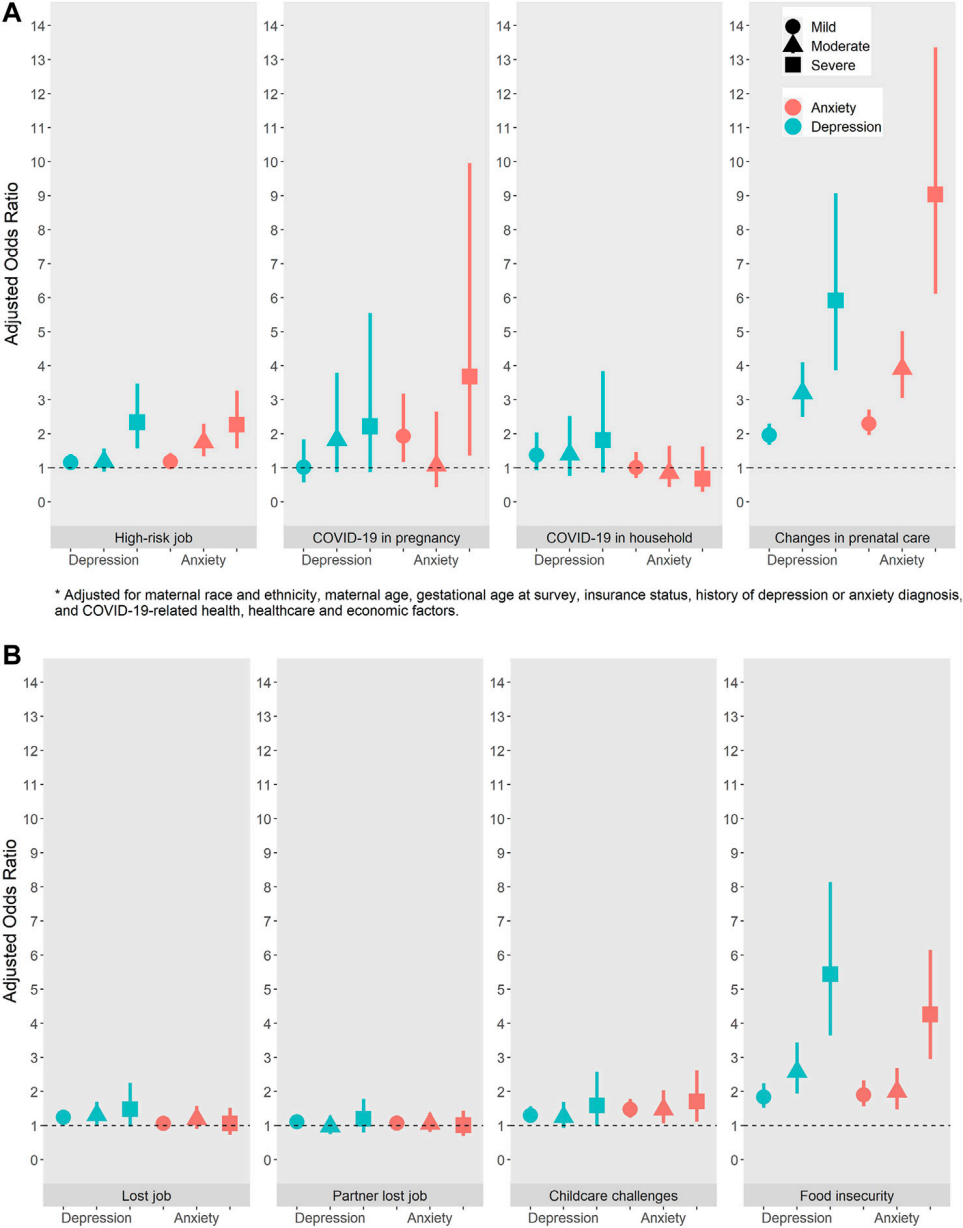
**(A)** Adjusted odds ratios for the relationship between COVID-19-related health and healthcare factors and prenatal depression (N = 6592) and anxiety (N = 6584) (California, United States, 2020). **(B)** Adjusted odds ratios for the relationship between COVID-19-related economic factors and prenatal depression (N = 6592) and anxiety (N = 6584) (California, United States, 2020).

Crude estimates are reported in Supplementary Tables S2A, S2B and multivariable adjusted estimates for the associations of COVID-19-related factors and depression and anxiety are reported in Figure 1A (depression) and Figure 1B (anxiety) and below.

#### Health and Healthcare Factors

Individuals who reported having COVID-19 during pregnancy had increased odds of anxiety symptoms [mild (aOR: 1.93, 95% CI: 1.17, 3.18), severe (aOR: 3.68, 95% CI: 1.36, 9.966)] compared to individuals without COVID-19 during pregnancy (Figure 1A). Increased odds of moderate and severe depression were also noted, but with less precise estimates. A non-significant elevated association was found between having a household member with COVID-19 and depression across severity but not anxiety. High-risk employment was associated with increased odds of severe depression (aOR: 2.34, 95% CI:1.57, 3.48), and moderate and severe anxiety moderate (aOR: 1.75, 95% CI: 1.34, 2.29), severe (aOR: 2.27, 95% CI: 1.57, 3.27). Distress due to changes in prenatal care was significantly associated with higher odds of depression and anxiety with the largest effect estimates at the moderate and severe levels [depression: mild (aOR: 1.96, 95% CI:1.68, 2.29), moderate (aOR: 3.2, 95% CI: 2.5, 4.1) and severe (aOR: 5.92, 95% CI: 3.87, 9.06); anxiety: mild (aOR: 2.3, 95% CI:1.96, 2.71), moderate (aOR: 3.91, 95% CI: 3.05, 5.02) and severe (aOR: 9.04, 95% CI: 6.12, 13.36)].

#### Economic Factors

Individuals who lost their job had higher odds of depression, but not anxiety compared to individuals who did not lose their job; however, the estimate for severe depression was less precise [depression: mild (aOR: 1.24, 95% CI:1.06, 1.46), moderate (aOR: 1.31, 95% CI: 1.01, 1.7) and severe (aOR: 1.48, 95% CI: 0.97, 2.25)] (Figure 1B). There was no association between a partner losing their job and the woman’s depression or anxiety. Individuals with COVID-19-related childcare challenges had increased odds of depression compared to individuals who did not have childcare impacted by COVID-19 [mild (aOR: 1.30, 95% CI: 1.09, 1.56), moderate (aOR: 1.25, 95% CI: 0.93, 1.69) and severe (aOR: 1.59, 95% CI: 0.99, 2.58) and anxiety (mild (aOR: 1.48, 95% CI: 1.23, 1.78), moderate (aOR: 1.47, 95% CI: 1.06, 2.69) and severe (aOR: 1.71, 95% CI: 1.11, 2.62)]. Although, the findings for the association between childcare challenges and depression were less precise. Food insecurity was significantly associated with both depression and anxiety with the association doubling between moderate and severe levels of depression and anxiety [depression: mild (aOR: 1.84, 95% CI: 1.52, 2.24), moderate (aOR: 2.58, 95% CI: 1.94, 3.44) and severe (aOR: 5.44, 95% CI: 3.64, 8.14); anxiety: mild (aOR: 1.90, 95% CI: 1.56, 2.32), moderate (aOR: 1.99, 95% CI: 1.47, 2.69) and severe (aOR: 4.26, 95% CI: 2.95, 6.15)].

## Discussion

This cross-sectional study examined the associations of several COVID-19 pandemic-related health, healthcare and economic factors with depression and anxiety symptoms in pregnant individuals during the first 6 months of the COVID-19 pandemic and height of stay-at-home orders. More severe symptoms of prenatal depression and/or anxiety were associated with self-report of COVID-19, employment increasing the risk of COVID-19, distress over changes in prenatal care, job loss, childcare challenges, and food insecurity. This study evaluated a larger number of COVID-19-related factors than previously assessed. In addition, previous research has not assessed the impact of COVID-19-related factors on prenatal depression and anxiety symptom severity, which is important for identifying those at greatest risk.

Elucidating risk factors for adverse mental health during pregnancy is of extreme significance as prenatal depression and anxiety can have detrimental impacts on the health of both mother and child. For example, individuals with prenatal depression and anxiety are at increased risk for unhealth maternal behaviors (e.g., diminished self-care, substance use) (10, 13–16, 27–29), preterm birth, low, birthweight, small-for-gestational age (SGA) neonates (10, 14, 29), and emotional and behavioral problems in the offspring (27, 28). Heightened prenatal depression and anxiety symptoms also increase the risk of postpartum depression (30, 31). Maternal self-harm and suicide is the leading cause of deaths in postpartum individuals (13, 16). Identifying factors that increase the risk of prenatal depression and anxiety may help develop targeted interventions to improve the health of pregnant individuals and their children.

### COVID-19-Related Health and Healthcare Factors

Our finding of the associations of self-reported COVID-19 and employment in a job with high-risk of COVID-19 exposure with prenatal depression and anxiety severity is consistent with other reports in pregnant individuals (32) and other populations (33, 34). In general, people with high-risk employment report more fatigue, health worries and fear (35). These findings support consideration of high-risk employment as a risk factor for mental health disorders in pregnancy, for use in depression/anxiety screening strategies.

We documented escalating severity of depression and anxiety symptoms associated with distress over COVID-19-related changes in prenatal care. These findings are similar to other studies documenting COVID-19-related changes in prenatal care and greater psychological distress (33, 36–38). The COVID-19 pandemic forced rapid implementation of hybrid models of care that were developing prior to the pandemic. Provider and healthcare system recognition of the psychological distress associated with changes to prenatal care should consider this when evaluating which care-delivery modifications to implement as standard prenatal care post pandemic or in future public health emergencies.

### COVID-19 Related Economic Factors

Previous research on the association of job loss or loss of source of income due to COVID-19 with prenatal depression and anxiety has reported mixed results (32, 33, 36, 38, 39). Our study suggested a relationship between participant’s job loss and depression, but not anxiety, while partner’s job loss was not related to psychological distress. However, food insecurity was strongly associated with prenatal depression and anxiety. This relationship between food insecurity and prenatal depression has been previously documented in research conducted prior to the pandemic (40, 41), yet the magnitude reported in our study (over 5-fold odds of severe depression associated with food insecurity) exceeds pre-COVID-19 findings (4-fold odds) (40). To our knowledge this is the first time that food insecurity has been studied in relation to prenatal anxiety. The strong relationship between food insecurity and psychological distress has important implications for clinical guidelines and health policy related to pregnancy in general, not just limited to times of public health emergencies. Addressing food insecurity may have a significant impact on the mental health of pregnant individuals, in addition to nutritional health. Given that not all food insecure individuals are eligible for government assistance, research into the effectiveness of non-governmental forms of food assistance, such as food banks and nutritional educational programs, is needed. Healthcare systems can also help by screening pregnant individuals for food insecurity and directing individuals to available resources.

Changes in childcare was also significantly related to increased odds of depression and anxiety. Other studies of pregnant individuals during the COVID-19 pandemic have documented similar findings (32, 38) highlighting the importance of public policy providing monetary support and childcare options especially during times of crises.

### Limitations

The low survey response rate raises concerns about non-response bias. However, our analyses were weighted to account for these differences. It is possible individuals with higher psychological distress were less likely to participate in the study and may explain why the prevalence of depression and anxiety was lower in our sample than has been reported in some, but not all (4), samples of pregnant individuals during the COVID-19 pandemic. However, we found that individuals who completed the survey were slightly more likely to have a history of depression or anxiety than individuals who did not complete the survey. Additionally, the cross-sectional study design limits our ability to establish causation. However, the likelihood of reverse causation is diminished by two points. It is unlikely that depression and anxiety led to any of the COVID-19-related factors with the exception of losing a job. Additionally, the mental health screeners asked about symptoms in the past 2 weeks while the time frame for the COVID-19-related factors reflected anytime since the start of the pandemic. Further, the depression and anxiety outcomes are based on symptoms in the past 2 weeks. If the COVID-19 related exposures illicit acute symptoms and the exposures occurred earlier in pregnancy, our findings may be an underestimate of the true association. Although this study sample represents a racially, ethnically, and socio-economically diverse population representative of Northern California, generalizability to other populations may be limited.

### Strengths

While there have been several studies on the mental health of pregnant individuals during the COVID-19 pandemic, few studies have contextualized the impact of COVID-19-related factors on prenatal depression and anxiety. Additional strengths include the study’s large sample size, diversity across sociodemographic characteristics, stages in pregnancy, and capture of COVID-19-related factors and psychological distress during the first 6 months of the pandemic and height of stay-at-home orders.

### Conclusion

This study contributes to the growing body of research documenting the adverse impact of public health emergencies experienced during pregnancy on maternal mental health (42–45). Findings from this study suggest that the COVID-19 pandemic may have severe mental health repercussions for pregnant individuals, an extremely vulnerable population. Several COVID-19-related health, healthcare and economic factors were found to adversely impact the mental health of pregnant individuals. These findings provide valuable information for the rapid development of policy, clinical and healthcare system interventions to promote the mental health of pregnant individuals as the COVID-19 pandemic continues and to effectively prepare for future pandemics. These study findings may also have broad policy and clinical implications outside of the US. The known short- and long-term consequences associated with prenatal psychological distress highlight the need for support services for pregnant individuals experiencing these COVID-19 related stressors. Healthcare systems should consider screening for identified stressors associated with poor mental health when conducting depression and anxiety screening and provide the appropriate interventions. Counseling services, social support and stress-reduction interventions to promote maternal mental health should be implemented. Additionally, continued postpartum monitoring of individuals who had high prenatal depression and anxiety symptoms during the COVID-19 pandemic and their children and appropriate interventions are important for decreasing the risk of the known adverse maternal and child outcomes associated with poor prenatal mental health.

## References

[1] CDC. COVID. Data Tracker: Centers for Disease Control and Prevention (2021). updated August 23, 2021. Available at: https://covid.cdc.gov/covid-data-tracker/#datatracker-home Accessed: August 10, 2021.

[2] Qin Z Shi L Xue Y Lin H Zhang J Liang P Prevalence and Risk Factors Associated with Self-Reported Psychological Distress Among Children and Adolescents during the COVID-19 Pandemic in China. JAMA Netw Open (2021) 4(1):e2035487. 10.1001/jamanetworkopen.2020.35487 33496797 PMC7838937

[3] Duan L Zhu G. Psychological Interventions for People Affected by the COVID-19 Epidemic. The Lancet Psychiatry (2020) 7(4):300–2. 10.1016/s2215-0366(20)30073-0 32085840 PMC7128328

[4] Iyengar U Jaiprakash B Haitsuka H Kim S. One Year into the Pandemic: A Systematic Review of Perinatal Mental Health Outcomes during COVID-19. Front Psychiatry (2021) 12(845):674194. 10.3389/fpsyt.2021.674194 34248710 PMC8264436

[5] ACOG. Coronavirus. COVID-19), Pregnancy, and Breastfeeding: A Message for Patients: American College of Obstetricians and Gynecologists (2021). updated August 4, 2021. Available at: https://www.acog.org/womens-health/faqs/coronavirus-covid-19-pregnancy-and-breastfeeding Accessed: August 10, 2021.

[6] Schumaker E. Here Are the States that Have Shut Down Nonessential Businesses. ABC News [Internet] (2020). Available at: https://abcnews.go.com/Health/states-shut-essential-businesses-map/story?id=69770806 Accessed: August 10, 2021.

[7] Trading Economics. United States Unemployment Rate: Trading Economics (2021). Available at: https://tradingeconomics.com/united-states/unemployment-rate Accessed: August 10, 2021.

[8] Witteveen D Velthorst E. Economic Hardship and Mental Health Complaints during COVID-19. Proc Natl Acad Sci U.S.A (2020) 117(44):27277–84. 10.1073/pnas.2009609117 33046648 PMC7959574

[9] Niles MT Bertmann F Belarmino EH Wentworth T Biehl E Neff R. The Early Food Insecurity Impacts of COVID-19. Nutrients (2020) 12(7):96. 10.3390/nu12072096 PMC7400862 32679788

[10] Grote NK Bridge JA Gavin AR Melville JL Iyengar S Katon WJ. A Meta-Analysis of Depression during Pregnancy and the Risk of Preterm Birth, Low Birth Weight, and Intrauterine Growth Restriction. Arch Gen Psychiatry (2010) 67(10):1012–24. 10.1001/archgenpsychiatry.2010.111 20921117 PMC3025772

[11] Accortt EE Cheadle ACD Dunkel Schetter C. Prenatal Depression and Adverse Birth Outcomes: an Updated Systematic Review. Matern Child Health J (2015) 19(6):1306–37. 10.1007/s10995-014-1637-2 25452215 PMC4447551

[12] Skurtveit S Selmer R Roth C Hernandez-Diaz S Handal M. Prenatal Exposure to Antidepressants and Language Competence at Age Three: Results from a Large Population-Based Pregnancy Cohort in Norway. Bjog: Int J Obstet Gy (2014) 121(13):1621–31. 10.1111/1471-0528.12821 24726047

[13] Mangla K Hoffman MC Trumpff C O’Grady S Monk C. Maternal Self-Harm Deaths: an Unrecognized and Preventable Outcome. Am J Obstet Gynecol (2019) 221(4):295–303. 10.1016/j.ajog.2019.02.056 30849358

[14] Venkatesh KK Riley L Castro VM Perlis RH Kaimal AJ. Association of Antenatal Depression Symptoms and Antidepressant Treatment with Preterm Birth. Obstet Gynecol (2016) 127(5):926–33. 10.1097/aog.0000000000001397 27054941 PMC10034858

[15] Young-Wolff KC Sarovar V Tucker L-Y Goler NC Alexeeff SE Ridout KK Association of Depression, Anxiety, and Trauma with Cannabis Use during Pregnancy. JAMA Netw Open (2020) 3(2):e1921333. 10.1001/jamanetworkopen.2019.21333 32074285 PMC7098171

[16] Goldman-Mellor S Margerison CE. Maternal Drug-Related Death and Suicide Are Leading Causes of Postpartum Death in California. Am J Obstet Gynecol (2019) 221(5):489. e9. 10.1016/j.ajog.2019.05.045 31173749 PMC6829056

[17] Gordon N Lin T. The Kaiser Permanente Northern California Adult Member Health Survey. Perm J (2016) 20(4):15–225. 10.7812/TPP/15-225 PMC5101088 27548806

[18] Kroenke K Spitzer RL Williams JBW. The PHQ-9. J Gen Intern Med (2001) 16(9):606–13. 10.1046/j.1525-1497.2001.016009606.x 11556941 PMC1495268

[19] Smith MV Gotman N Lin H Yonkers KA. Do the PHQ-8 and the PHQ-2 Accurately Screen for Depressive Disorders in a Sample of Pregnant Women? Gen Hosp Psychiatry (2010) 32(5):544–8. 10.1016/j.genhosppsych.2010.04.011 20851275 PMC2943487

[20] Kroenke K Strine TW Spitzer RL Williams JB Berry JT Mokdad AH. The PHQ-8 as a Measure of Current Depression in the General Population. J Affect Disord (2009) 114(1):163–73. 10.1016/j.jad.2008.06.026 18752852

[21] Spitzer RL Kroenke K Williams JBW Löwe B. A Brief Measure for Assessing Generalized Anxiety Disorder. Arch Intern Med (2006) 166(10):1092–7. 10.1001/archinte.166.10.1092 16717171

[22] Simpson W Glazer M Michalski N Steiner M Frey BN. Comparative Efficacy of the Generalized Anxiety Disorder 7-item Scale and the Edinburgh Postnatal Depression Scale as Screening Tools for Generalized Anxiety Disorder in Pregnancy and the Postpartum Period. Can J Psychiatry (2014) 59(8):434–40. 10.1177/070674371405900806 25161068 PMC4143300

[23] Ames JL Ferrara A Avalos LA Badon SE Greenberg MB Hedderson MM COVID-19 Prevalence, Symptoms, and Sociodemographic Disparities in Infection Among Insured Pregnant Women in Northern California. PLOS ONE (2021) 16(9):e0256891. 10.1371/journal.pone.0256891 34478463 PMC8415576

[24] Gundersen C Engelhard EE Crumbaugh AS Seligman HK. Brief Assessment of Food Insecurity Accurately Identifies High-Risk US Adults. Public Health Nutr (2017) 20(8):1367–71. 10.1017/s1368980017000180 28215190 PMC10261547

[25] White IR Royston P Wood AM. Multiple Imputation Using Chained Equations: Issues and Guidance for Practice. Statist Med (2011) 30(4):377–99. 10.1002/sim.4067 21225900

[26] Rubin DB. Multiple Imputation for Nonresponse in Surveys. New York: John Wiley & Sons (1987).

[27] Gentile S. Untreated Depression during Pregnancy: Short- and Long-Term Effects in Offspring. A Systematic Review. Neuroscience (2017) 342:154–66. 10.1016/j.neuroscience.2015.09.001 26343292

[28] Goodman SH Cullum KA Dimidjian S River LM Kim CY. Opening Windows of Opportunities: Evidence for Interventions to Prevent or Treat Depression in Pregnant Women Being Associated with Changes in Offspring's Developmental Trajectories of Psychopathology Risk. Dev Psychopathol (2018) 30(3):1179–96. 10.1017/s0954579418000536 30068424

[29] Jarde A Morais M Kingston D Giallo R MacQueen GM Giglia L Neonatal Outcomes in Women with Untreated Antenatal Depression Compared with Women without Depression. JAMA Psychiatry (2016) 73(8):826–37. 10.1001/jamapsychiatry.2016.0934 27276520

[30] Gavin NI Gaynes BN Lohr KN Meltzer-Brody S Gartlehner G Swinson T. Perinatal Depression: a Systematic Review of Prevalence and Incidence. Obstet Gynecol (2005) 106(5 Pt 1):1071–83. 10.1097/01.AOG.0000183597.31630.db 16260528

[31] Gaynes BN Gavin N Meltzer-Brody S Lohr KN Swinson T Gartlehner G Perinatal Depression: Prevalence, Screening Accuracy, and Screening Outcomes. Evid Rep Technol Assess (Summ) (2005)(119) 1–8. 10.1037/e439372005-001 PMC4780910 15760246

[32] Moyer CA Compton SD Kaselitz E Muzik M. Pregnancy-related Anxiety during COVID-19: a Nationwide Survey of 2740 Pregnant Women. Arch Womens Ment Health (2020) 23(6):757–65. 10.1007/s00737-020-01073-5 32989598 PMC7522009

[33] Lebel C MacKinnon A Bagshawe M Tomfohr-Madsen L Giesbrecht G. Elevated Depression and Anxiety Symptoms Among Pregnant Individuals during the COVID-19 Pandemic. J Affective Disord (2020) 277:5–13. 10.1016/j.jad.2020.07.126 PMC7395614 32777604

[34] Shi J Gao Y Zhao L Li Y Yan M Niu MM Prevalence of Delirium, Depression, Anxiety, and post-traumatic Stress Disorder Among COVID-19 Patients: Protocol for a Living Systematic Review. Syst Rev (2020) 9(1):258. 10.1186/s13643-020-01507-2 33158456 PMC7646715

[35] Brooks SK Dunn R Amlôt R Rubin GJ Greenberg N. A Systematic, Thematic Review of Social and Occupational Factors Associated with Psychological Outcomes in Healthcare Employees during an Infectious Disease Outbreak. J Occup Environ Med (2018) 60(3):248–57. 10.1097/jom.0000000000001235 29252922

[36] Preis H Mahaffey B Heiselman C Lobel M. Vulnerability and Resilience to Pandemic-Related Stress Among U.S. Women Pregnant at the Start of the COVID-19 Pandemic. Soc Sci Med (2020) 266:113348. 10.1016/j.socscimed.2020.113348 32927382 PMC7474815

[37] Bo H-X Yang Y Chen J Zhang M Li Y Zhang D-Y Prevalence of Depressive Symptoms Among Pregnant and Postpartum Women in China during the COVID-19 Pandemic. Psychosom Med (2021) 83(4):345–50. 10.1097/psy.0000000000000904 33337594

[38] Basu A Kim HH Basaldua R Choi KW Charron L Kelsall N A Cross-National Study of Factors Associated with Women's Perinatal Mental Health and Wellbeing during the COVID-19 Pandemic. PLoS One (2021) 16(4):e0249780. 10.1371/journal.pone.0249780 33882096 PMC8059819

[39] Liu JHP Alberg AJ Hair NL Whitaker KM Simon J Taylor SK. Mental Health Among Pregnant Women with COVID-19-Related Stressors and Worries in the United States. Birth (2021) 48(4):470–9. 10.1111/birt.12554 34008216 PMC8239832

[40] Richards M Weigel M Li M Rosenberg M Ludema C. Household Food Insecurity and Antepartum Depression in the National Children's Study. Ann Epidemiol (2020) 44:38–44. 10.1016/j.annepidem.2020.01.010 32220512

[41] Laraia BA Siega-Riz AM Gundersen C Dole N. Psychosocial Factors and Socioeconomic Indicators Are Associated with Household Food Insecurity Among Pregnant Women. J Nutr (2006) 136(1):177–82. 10.1093/jn/136.1.177 16365079

[42] Huizink AC Bartels M Rose RJ Pulkkinen L Eriksson CJP Kaprio J. Chernobyl Exposure as Stressor during Pregnancy and Hormone Levels in Adolescent Offspring. J Epidemiol Community Health (2008) 62(4):e5. 10.1136/jech.2007.060350 18365332 PMC2562331

[43] Ehrlich M Harville E Xiong X Buekens P Pridjian G Elkind-Hirsch K. Loss of Resources and hurricane Experience as Predictors of Postpartum Depression Among Women in Southern Louisiana. J Women's Health (2010) 19(5):877–84. 10.1089/jwh.2009.1693 PMC2875990 20438305

[44] Xiong X Harville EW Mattison DR Elkind-Hirsch K Pridjian G Buekens P. Hurricane Katrina Experience and the Risk of post-traumatic Stress Disorder and Depression Among Pregnant Women. Am J Disaster Med (2010) 5(3):181–7. 10.5055/ajdm.2010.0020 20701175 PMC3501144

[45] Lee DTS Sahota D Leung TN Yip ASK Lee FFY Chung TKH. Psychological Responses of Pregnant Women to an Infectious Outbreak: a Case-Control Study of the 2003 SARS Outbreak in Hong Kong. J Psychosomatic Res (2006) 61(5):707–13. 10.1016/j.jpsychores.2006.08.005 PMC7094779 17084150

