# Supplementary material for: Associations of COVID-19-Related Health, Healthcare and Economic Factors With Prenatal Depression and Anxiety
Source: Int J Public Health. 2022 May 4;67:1604433. doi: 10.3389/ijph.2022.1604433 (PMC9114304; doi:10.3389/ijph.2022.1604433)
Supplement: Supplementary file 1 [file Table1.DOCX]

| **Supplemental Table 1. Characteristics of pregnant individuals with completed surveys compared to the overall invited population of pregnant individuals (California, United States, 2020)** | | | |
| --- | --- | --- | --- |
|  | **All individuals invited**  **(n=31,299)** | **Completed the survey**  **(n=6,628)** | **Did not complete the survey**  **(n=24,671)** |
| **Sociodemographic characteristics** |  |  |  |
| Age at survey invitation (years), mean (SD) | 31 (5) | 32 (5) | 31 (5) |
| Missing | 209 | 2 | 207 |
| **Race/ethnicity, n (%)** |  |  |  |
| White | 10,887 (37) | 3236 (51) | 7651 (33) |
| Asian | 8510 (29) | 1337 (21) | 6021 (26) |
| Hispanic | 7479 (25) | 1458 (23) | 7173 (31) |
| Black | 2289 (8) | 244 (4) | 2045 (9) |
| Other | 602 (2) | 104 (2) | 2045 (9) |
| Missing | 1532 | 249 | 1283 |
| **Medicaid insurance, n (%)** | 3685 (9) | 316 (5) | 2369 (10) |
| Missing | 576 | 59 | 517 |
| **Neighborhood deprivation index category, n (%)** |  |  |  |
| Quartile 1 | 7499 (25) | 2014 (31) | 5435 (23) |
| Quartile 2 | 7543 (24) | 1803 (28) | 5740 (24) |
| Quartile 3 | 7744 (25) | 1526 (23) | 6218 (26) |
| Quartile 4 | 7857 (26) | 1212 (18) | 6645 (28) |
| Missing | 706 | 73 | 633 |
| **Parity** |  |  |  |
| 0 | 9731 (31) | 2458 (37) | 7273 (29) |
| 1 | 12615 (40) | 2751 (42) | 9864 (40) |
| 2+ | 6244 (20) | 1065 (16) | 5179 (21) |
| Missing | 2709 (9) | 354 (5) | 2355 (10) |
| **History of mood disorder** |  |  |  |
| Yes | 6984 (22) | 1606 (24) | 5378 (22) |
| No | 24315 (78) | 5022 (76) | 19293 (78) |
| **Trimester at survey completion, n (%)** |  |  |  |
| First trimester (2-13 weeks) | 8110 (26) | 816 (12) | 7294 (30) |
| Second trimester (14-26 weeks) | 15,602 (50) | 3895 (59) | 11,707 (47) |
| Third trimester (27-42 weeks) | 7587 (24) | 1917 (29) | 5670 (23) |
