# Supplementary material for: Associations of COVID-19-Related Health, Healthcare and Economic Factors With Prenatal Depression and Anxiety
Source: Int J Public Health. 2022 May 4;67:1604433. doi: 10.3389/ijph.2022.1604433 (PMC9114304; doi:10.3389/ijph.2022.1604433)
Supplement: Supplementary file 2 [file Table2.docx]

| **Supplemental Table 2a. Crude Odds Ratio (cOR) for the association with COVID-19-related health, healthcare and economic factors and prenatal depression severity (N=6592) (California, United States, 2020).** | | | | |
| --- | --- | --- | --- | --- |
|  | **Depression** | | | |
|  | **None** | **Mild** | **Moderate** | **Severe** |
|  |  | OR (95% CI) | OR (95% CI) | OR (95% CI) |
| **COVID-19-related Factors** |  |  |  |  |
| **Health and Healthcare** |  |  |  |  |
| **COVID-19 in pregnancy** |  |  |  |  |
| Yes |  |  |  |  |
| No | ref | 1.09 (0.79,2.05) | 2.31 (0.75,2.38) | 3.75 (0.7,2.95) |
| **Household member had COVID-19** | ref | ref | ref | ref |
| Yes |  |  |  |  |
| No | ref | 1.45 (0.87,1.56) | 1.75 (0.81,1.94) | 2.67 (0.76,2.37) |
| **High-risk job** | ref | ref | ref | ref |
| Yes |  |  |  |  |
| No | ref | 1.43 (0.93,1.26) | 1.72 (0.89,1.43) | 3.71 (0.86,1.61) |
| **Distress due to prenatal care changes** | ref | ref | ref | ref |
| Moderately/Extremely |  |  |  |  |
| Mildly/Not at all | ref | 2.19 (1.92,2.5) | 3.8 (3.08,4.69) | 7.97 (5.63,11.28) |
| **Economic Factors** | ref | ref | ref | ref |
| **Lost Job** |  |  |  |  |
| Yes |  |  |  |  |
| No | ref | 1.4 (0.94,1.23) | 1.58 (0.9,1.39) | 2.3 (0.86,1.61) |
| **Partner lost job** | ref | ref | ref | ref |
| Yes |  |  |  |  |
| No | ref | 1.26 (0.94,1.23) | 1.34 (0.9,1.39) | 2.09 (0.86,1.61) |
| **Childcare challenges** | ref | ref | ref | ref |
| Yes |  |  |  |  |
| No | ref | 1.22 (1.07,1.38) | 1.35 (1.1,1.65) | 1.49 (1.09,2.05) |
| **Food Insecurity** | ref | ref | ref | ref |
| Yes |  |  |  |  |
| No | ref | 2.17 (1.85,2.55) | 3.93 (3.16,4.9) | 9.78 (7.17,13.32) |
|  | ref | ref | ref | ref |

| **Supplemental Table 2b. Crude Odds Ratio (cOR) for the association with COVID-19-related health, healthcare and economic factors and prenatal anxiety severity (N=6584), (California, United States, 2020).** | | | | |
| --- | --- | --- | --- | --- |
|  | **Anxiety** | | | |
|  | **None** | **Mild** | **Moderate** | **Severe** |
|  |  | OR (95% CI) | OR (95% CI) | OR (95% CI) |
| **COVID-19-related Factors** |  |  |  |  |
| **Health and Healthcare** |  |  |  |  |
| **COVID-19 in pregnancy** |  |  |  |  |
| Yes | ref | 1.71 (0.81,1.92) | 1.31 (0.69,3.08) | 2.87 (0.69,3.11) |
| No | ref | ref | ref | ref |
| **Household member had COVID-19** |  |  |  |  |
| Yes | ref | 1.29 (0.86,1.57) | 1.22 (0.79,2.09) | 1.39 (0.73,2.69) |
| No | ref | ref | ref | ref |
| **High-risk job** |  |  |  |  |
| Yes | ref | 1.34 (0.92,1.28) | 2.42 (0.9,1.4) | 3.35 (0.86,1.56) |
| No | ref | ref | ref | ref |
| **Distress due to prenatal care changes** |  |  |  |  |
| Moderately/Extremely | ref | 2.42 (2.11,2.78) | 4.87 (3.95,6.01) | 10.04 (7.22,13.96) |
| Mildly/Not at all | ref | ref | ref | ref |
| **Economic Factors** |  |  |  |  |
| **Lost Job** |  |  |  |  |
| Yes | ref | 1.25 (0.93,1.24) | 1.47 (0.9,1.39) | 1.89 (0.86,1.57) |
| No | ref | ref | ref | ref |
| **Partner lost job** |  |  |  |  |
| Yes | ref | 1.3 (0.93,1.23) | 1.48 (0.9,1.39) | 1.94 (0.87,1.56) |
| No | ref | ref | ref | ref |
| **Childcare challenges** |  |  |  |  |
| Yes | ref | 1.43 (1.25,1.62) | 1.17 (0.95,1.46) | 1.78 (1.33,2.37) |
| No | ref | ref | ref | ref |
| **Food Insecurity** |  |  |  |  |
| Yes | ref | 2.06 (1.75,2.43) | 3.44 (2.75,4.31) | 7.31 (5.49,9.72) |
| No | ref | ref | ref | ref |
